# Hyperfibrinolysis in severe isolated traumatic brain injury may occur without tissue hypoperfusion: a retrospective observational multicentre study

**DOI:** 10.1186/s13054-017-1811-1

**Published:** 2017-08-23

**Authors:** Mineji Hayakawa, Kunihiko Maekawa, Shigeki Kushimoto, Hiroshi Kato, Junichi Sasaki, Hiroshi Ogura, Tetsuya Matsuoka, Toshifumi Uejima, Naoto Morimura, Hiroyasu Ishikura, Akiyoshi Hagiwara, Munekazu Takeda, Naoyuki Kaneko, Daizoh Saitoh, Daisuke Kudo, Takashi Kanemura, Takayuki Shibusawa, Shintaro Furugori, Yoshihiko Nakamura, Atsushi Shiraishi, Kiyoshi Murata, Gou Mayama, Arino Yaguchi, Shiei Kim, Osamu Takasu, Kazutaka Nishiyama

**Affiliations:** 1 Emergency and Critical Care Center, Hokkaido University Hospital, N14W5, Kita-ku, Sapporo, 060-8648 Japan; 2 Division of Emergency and Critical Care Medicine, Tohoku University Graduate School of Medicine, Sendai, Japan; 3 Department of Critical Care and Traumatology, National Hospital Organization Disaster Medical Center, Tokyo, Japan; 4 Department of Emergency & Critical Care Medicine, Keio University School of Medicine, Tokyo, Japan; 5 Department of Traumatology and Acute Critical Medicine, Osaka University Graduate School of Medicine, Osaka, Japan; 6 Senshu Trauma and Critical Care Center, Rinku General Medical Center, Osaka, Japan; 7 Department of Emergency and Critical Care Medicine, Kinki University Faculty of Medicine, Osaka, Japan; 8 Department of Emergency and Critical Care Medicine, The University of Tokyo Hospital, Tokyo, Japan; 9 Department of Emergency and Critical Care Medicine, Faculty of Medicine, Fukuoka University, Fukuoka, Japan; 10 Department of Emergency Medicine and Critical Care, National Center for Global Health and Medicine, Tokyo, Japan; 11 Department of Critical Care and Emergency Medicine, Tokyo Women’s Medical University, Tokyo, Japan; 12 Trauma and Emergency Center, Fukaya Red Cross Hospital, Saitama, Japan; 13 Division of Traumatology, Research Institute, National Defence Medical College, Saitama, Japan; 14 Department of Emergency Medicine, Yokohama City University Graduate School of Medicine, Yokohama, Japan; 15 Trauma and Acute Critical Care Medical Center, Tokyo Medical and Dental University Hospital of Medicine, Tokyo, Japan; 16 Department of Emergency & Critical Care Medicine, Nippon Medical School, Tokyo, Japan; 17 Department of Emergency and Critical Care Medicine, Kurume University School of Medicine, Kurume, Japan; 18 Department of Emergency and Critical Care Medicine, Juntendo University Urayasu Hospital, Chiba, Japan

**Keywords:** Coagulopathy, Disseminated intravascular coagulation, Hypoperfusion, Hyperfibrinolysis, Traumatic brain injury

## Abstract

**Background:**

Hyperfibrinolysis is a critical complication in severe trauma. Hyperfibrinolysis is traditionally diagnosed via elevated D-dimer or fibrin/fibrinogen degradation product levels, and recently, using thromboelastometry. Although hyperfibrinolysis is observed in patients with severe isolated traumatic brain injury (TBI) on arrival at the emergency department (ED), it is unclear which factors induce hyperfibrinolysis. The present study aimed to investigate the factors associated with hyperfibrinolysis in patients with isolated severe TBI.

**Methods:**

We conducted a multicentre retrospective review of data for adult trauma patients with an injury severity score ≥ 16, and selected patients with isolated TBI (TBI group) and extra-cranial trauma (non-TBI group). The TBI group included patients with an abbreviated injury score (AIS) for the head ≥ 4 and an extra-cranial AIS < 2. The non-TBI group included patients with an extra-cranial AIS ≥ 3 and head AIS < 2. Hyperfibrinolysis was defined as a D-dimer level ≥ 38 mg/L on arrival at the ED. We evaluated the relationships between hyperfibrinolysis and injury severity/tissue injury/tissue perfusion in TBI patients by comparing them with non-TBI patients.

**Results:**

We enrolled 111 patients in the TBI group and 126 in the non-TBI group. In both groups, patients with hyperfibrinolysis had more severe injuries and received transfusion more frequently than patients without hyperfibrinolysis. Tissue injury, evaluated on the basis of lactate dehydrogenase and creatine kinase levels, was associated with hyperfibrinolysis in both groups. Among patients with TBI, the mortality rate was higher in those with hyperfibrinolysis than in those without hyperfibrinolysis. Tissue hypoperfusion, evaluated on the basis of lactate level, was associated with hyperfibrinolysis in only the non-TBI group. Although the increase in lactate level was correlated with the deterioration of coagulofibrinolytic variables (prolonged prothrombin time and activated partial thromboplastin time, decreased fibrinogen levels, and increased D-dimer levels) in the non-TBI group, no such correlation was observed in the TBI group.

**Conclusions:**

Hyperfibrinolysis is associated with tissue injury and trauma severity in TBI and non-TBI patients. However, tissue hypoperfusion is associated with hyperfibrinolysis in non-TBI patients, but not in TBI patients. Tissue hypoperfusion may not be a prerequisite for the occurrence of hyperfibrinolysis in patients with isolated TBI.

**Electronic supplementary material:**

The online version of this article (doi:10.1186/s13054-017-1811-1) contains supplementary material, which is available to authorized users.

## Background

The acute phase of severe trauma is a frequent setting for trauma-induced coagulopathy, which may develop into severe haemorrhage due to coagulation abnormalities and contribute to a poor outcome [1–11]. Hyperfibrinolysis is one of the distinctive features of trauma-induced coagulopathy [1–11]. Hyperfibrinolysis has been traditionally recognized by the presence of elevated D-dimer or fibrin/fibrinogen degradation product levels [2–6]. In addition, recent studies have reported that hyperfibrinolysis is detected as clot lysis on thromboelastometry [7–11]. In thromboelastometry, clot lysis is observed when the α_2_-antiplasmin cannot inhibit the action of free plasmin activated by tissue-plasminogen activator (t-PA) in blood samples [9]. On the other hand, elevation of the D-dimer level results from degradation of fibrin by plasmin, regardless of the marked increase in free t-PA level and consumption of α_2_-antiplasmin [12]. Therefore, elevated D-dimer levels may be a sensitive indicator of hyperfibrinolysis and a predictor of poor outcome in patients with severe trauma [3, 5, 9].

In patients with isolated severe traumatic brain injury (TBI), hyperfibrinolysis is often observed on arrival at the emergency department (ED), and is associated with intracranial haematoma enlargement [5, 6, 13–16]. Furthermore, hyperfibrinolysis contributes to the development of coagulopathy and a poor outcome in isolated severe TBI [5, 14–16]. Maegele proposed that hyperfibrinolysis was induced by tissue hypoperfusion in TBI [16]. Furthermore, Cohen at al. [17] reported that TBI alone does not cause hyperfibrinolysis and coagulopathy, but must be coupled with tissue hypoperfusion. Conversely, Lustenberger et al. [18] indicated that tissue hypoperfusion was not essential for the development of coagulation abnormalities in patients with severe TBI. Therefore, the relationship between tissue hypoperfusion and hyperfibrinolysis remains unclear in patients with isolated TBI.

As is the case with hypoperfusion, tissue injury is speculated to independently cause hyperfibrinolysis in severe trauma [1–3, 13, 14, 19, 20]. Immediately after trauma, microparticles exposing tissue factor [13, 19, 21–23] and damage-associated molecular patterns (DAMPs), such as histones [24, 25] and mitochondrial DNA [26], are released from damaged tissue into the systemic circulation and activate the coagulation system. The activation of the coagulation system triggers the activation of the fibrinolytic system, thus leading to the generation of plasmin [27]. Massive tissue injury induces excessive release of tissue factor and DAMPs and unregulated activation of the coagulation system [1, 3, 13, 14, 24–26], which induces increased plasmin generation and the consumption of alpha 2-plasmin inhibitor [13, 14, 20, 28]. Low levels of alpha 2-plasmin inhibitor may lead to accelerated fibrinolysis and result in hyperfibrinolysis [13, 14, 20, 28]. However, the pathophysiological role of tissue injury and hypoperfusion in the development of hyperfibrinolysis remains unclear in patients with and without TBI.

The present study aimed to investigate the factors associated with hyperfibrinolysis in patients with isolated severe TBI. We evaluated the relationships between hyperfibrinolysis and injury severity/tissue injury/tissue perfusion in patients with TBI by comparing them with non-TBI patients.

## Methods

### Patient selection and data collection

This retrospective study investigated coagulation disorders in patients with severe trauma at 15 tertiary emergency and critical care centres in Japan (Japanese Observational Study for Coagulation and Thrombolysis in Early Trauma, J-OCTET). The J-OCTET recruited consecutive patients with severe trauma with an injury severity score (ISS) ≥ 16, who were admitted to EDs from January 2012 through December 2012 for the purpose of investigating coagulation abnormalities, transfusion, and mortality [3, 29, 30]. Patients were excluded if they were younger than 18 years; pregnant; or exhibited cardiac arrest, burns, isolated cervical spine injury not caused by a high-energy accident, or liver cirrhosis. The laboratory test results, which were promptly obtained on arrival at the ED, clinical background, treatment, and outcome of the patients were retrospectively analysed.

In the present study, we selected patients with isolated TBI and patients with severe extra-cranial trauma without TBI from the J-OCTET database. Patients were excluded if: (1) they had used anti-coagulant or anti-platelet drugs before the accident; (2) they had received infusions before arrival in the ED; (3) they had penetrating trauma; or (4) the D-dimer level had not been measured on arrival at the ED.

### Definitions

We defined patients with isolated TBI (the TBI group) as patients with an abbreviated injury score (AIS) for the head ≥ 4 and an extra-cranial AIS < 2. Patients with isolated TBI and with a head AIS of 3 and extra-cranial AIS < 2 were not included, because the J-OCTET recruited only patients with severe trauma, with an ISS ≥ 16. Patients with severe extra-cranial trauma without TBI (the non-TBI group) were defined as patients with an extra-cranial AIS ≥ 3 and head AIS < 2. Hyperfibrinolysis was defined as a D-dimer level ≥ 38 mg/L on arrival at the ED, on the basis of our previous study, which indicated that a high D-dimer level was a strong predictor of poor outcome [3]. Lactate was used as a surrogate marker of tissue hypoperfusion, similar to previous studies [2, 7, 11]. Lactate dehydrogenase (LDH) and creatine kinase (CK) were used as surrogate markers of tissue injury, because they are expressed by brain tissue and muscle as well as various other tissues [31, 32]. The patient’s outcome was evaluated according to mortality rates at 24 hours, 48 hours, and 28 days, and at the time of hospital discharge (in-hospital mortality).

### Statistical analysis

All variables are expressed as the median and interquartile range (i.e., the first to third quartiles) or number (percentage). Intergroup comparisons were made using the Mann–Whitney *U* test or χ^2^ test. Multiple logistic regression analysis was used to assess relationships between hyperfibrinolysis and patient characteristics. Patient age and sex were used as covariates in the multiple logistic regression analysis. Correlations between two measurements were investigated via Pearson’s correlation analysis after logarithmic transformation. SPSS 22.0 J (SPSS Inc., Chicago, IL, USA) was used for all statistical analyses. The level of significance was set at *P* < 0.05; we did not conduct or adjust for multiple tests because of the retrospective, exploratory nature of this study.

## Results

### Patient characteristics

**Fig. 1:**
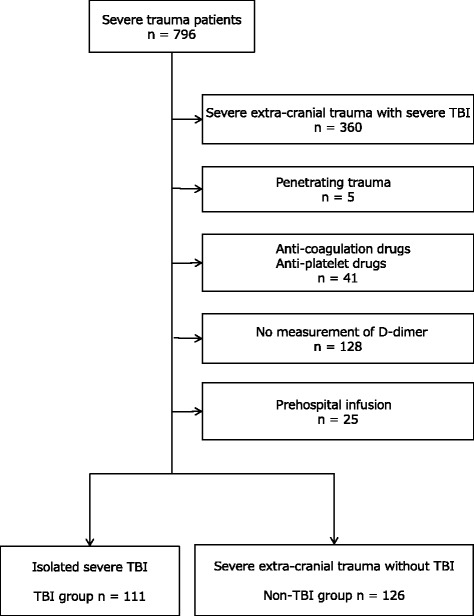
Flow chart of the selection of patients in the traumatic brain injury (TBI) and non-TBI groups. The Japanese Observational Study for Coagulation and Thrombolysis in Early Trauma (J-OCTET) recruited 796 patients with severe trauma with an injury severity score ≥ 16, who were admitted to the emergency department (ED). Patients with isolated severe TBI were defined as patients with an abbreviated injury score (AIS) for the head ≥ 4 and an extra-cranial AIS < 2. Patients with severe extra-cranial trauma without TBI (non-TBI patients) were defined by an extra-cranial AIS ≥ 3 and head AIS < 2. Patients were excluded if they had used anti-coagulant or anti-platelet drugs before the accident or received an infusion before arrival at the ED. Patients with penetrating trauma and those whose D-dimer levels were not measured on arrival at the ED were also excluded

The TBI group included 111 patients and the non-TBI group included 126 patients (Fig. 1). The patient characteristics in the TBI and non-TBI groups are shown in Table 1. The patients in the TBI group were older than those in the non-TBI group. The revised trauma scores in the TBI group were worse than those in the non-TBI group. Although many variables differed between the two groups, the D-dimer levels on admission to the ED were not different.

**Table 1:** Characteristics of the patients

	TBI n = 111	Non-TBI n = 126	*P* value
Age, years	64 (43–76)	44 (30–64)	<0.001
Male, *n* (%)	83 (75)	82 (65)	0.105
ISS	25 (17–26)	21 (17–27)	0.044
Revised trauma score	6.90 (5.97–7.84)	7.84 (7.11–7.84)	<0.001
Heart rate, per min	85 (74–93)	89 (76–103)	0.034
Systolic BP, mmHg	152 (129–178)	116 (96–138)	<0.001
Respiratory rate, per min	19 (18–24)	23 (18–28)	0.001
Body temperature, °C	36.2 (35.8–36.7)	36.4 (35.8–36.8)	0.334
Glasgow coma scale score	12 (6–14)	14 (13–15)	<0.001
Time from the accident to sample collection			
0–30 min, *n* (%)	33 (30)	29 (24)	0.122
31–60 min, *n* (%)	56 (51)	64 (52)	
61–90 min, *n* (%)	7 (6)	18 (15)	
91–120 min, *n* (%)	7 (6)	2 (2)	
Over 120 min, *n* (%)	6 (6)	10 (8)	
Unknown, *n* (%)	2 (2)	3 (2)	
Arterial blood gas analyses			
pH	7.39 (7.35–7.42)	7.38 (7.33–7.42)	0.061
Base deficit, mmol/L	1.2 (-0.4–3.0)	1.8 (0.2–4.4)	0.044
Lactate, mmol/L	2.40 (1.40–3.20)	2.65 (1.70–3.62)	0.081
Laboratory parameters			
White blood cells, × 10^9^/L	9.9 (7.3–12.8)	11.8 (9.2–16.0)	<0.001
Haemoglobin, g/dL	13.7 (12.4–14.8)	12.8 (11.3–14.4)	0.009
Platelet count, × 10^9^/L	18.3 (14.7–23.5)	22.9 (18.2–26.3)	<0.001
AST, U/L	31 (25–44)	76 (45–156)	<0.001
ALT, U/L	25 (17–34)	49 (29–100)	<0.001
LDH, U/L	270 (217–327)	469 (348–723)	<0.001
CK, U/L	149 (99–246)	317 (214–532)	<0.001
PT-INR	1.01 (0.96–1.10)	1.06 (0.99–1.13)	0.009
APTT, sec	25.0 (23.0–28.2)	25.6 (23.5–29.0)	0.306
Fibrinogen, mg/dL	246 (181–296)	229 (196–280)	0.569
D-dimer, μg/mL	22.4 (7.6–49.6)	23.8 (8.1–58.6)	0.500

*ISS* injury severity score, *BP* blood pressure, *AST* aspartate aminotransferase, *ALT* alanine aminotransferase, *LDH* lactate dehydrogenase, *CK* creatine kinase, *PT-INR* prothrombin time–international normalized ratio, *APTT* activated partial thromboplastin time

Table 2 presents the characteristics of patients with and without hyperfibrinolysis in each group. On arrival at the ED, 34 (31%) and 46 (37%) patients in the TBI and non-TBI groups, respectively, were diagnosed as having hyperfibrinolysis. The frequency of hyperfibrinolysis was not significantly different between the groups (*P* = 0.340). In both groups, the injury severity scores (ISS and revised trauma score) of the patients with hyperfibrinolysis were higher than those of patients without hyperfibrinolysis. Although the systolic blood pressure measurements in patients with TBI and hyperfibrinolysis were higher than those in patients with TBI and no hyperfibrinolysis, the systolic blood pressure measurements in non-TBI patients with hyperfibrinolysis were lower than those in non-TBI patients without hyperfibrinolysis. The levels of LDH and CK related to tissue injury in patients with hyperfibrinolysis were greater than those in patients without hyperfibrinolysis in both groups. The lactate levels did not differ between patients with and without hyperfibrinolysis in both groups.

**Table 2:** Characteristics of the patients with and without hyperfibrinolysis in each group

	TBI		*P* value	Non-TBI		*P* value
	Hyperfibrinolysis (-) *n* = 77	Hyperfibrinolysis (+) *n* = 34		Hyperfibrinolysis (-) *n* = 80	Hyperfibrinolysis (+) *n* = 46	
Age, years	61 (41–72)	70 (51–83)	0.028	45 (36–62)	43 (26–70)	0.895
Male, *n* (%)	60 (78)	23 (68)	0.251	61 (76)	25 (54)	0.001
ISS	17 (16–26)	25 (25–26)	0.011	20 (17–25)	27 (18–34)	0.003
Revised trauma score	7.10 (6.17–7.84)	5.50 (4.09–7.84)	<0.001	7.84 (7.55–7.84)	7.55 (6.61–7.84)	0.022
Heart rate, per min	85 (75–93)	84 (65–93)	0.467	85 (71–98)	96 (85–111)	0.001
Systolic BP, mmHg	144 (126–169)	170 (138–193)	0.004	122 (103–140)	104 (87–128)	0.016
Respiratory rate, per min	19 (18–23)	20 (16–25)	0.774	22 (18–25)	24 (20–30)	0.036
Body temperature, °C	36.2 (35.8–36.7)	36.2 (35.6–36.8)	0.708	36.5 (35.8–36.9)	36.3 (35.7–36.6)	0.210
Glasgow coma scale score	13 (9–14)	6 (3–13)	<0.001	14 (14–15)	14 (12–15)	0.017
Time from the accident to sample collection						
0–30 min, *n* (%)	21 (28)	12 (35)	0.469	17 (22)	12 (27)	0.641
31–60 min, *n* (%)	38 (51)	18 (53)		43 (54)	21 (48)	
61–90 min, *n* (%)	5 (7)	2 (6)		13 (17)	5 (11)	
91–120 min, *n* (%)	7 (9)	0 (0)		1 (1)	1 (2)	
Over 120 min, *n* (%)	4 (5)	2 (6)		5 (6)	5 (11)	
Unknown, *n* (%)	2 (3)	0 (0)		1 (1)	2 (4)	
Arterial blood gas analyses						
pH	7.39 (7.35–7.43)	7.39 (7.37–7.42)	0.824	7.38 (7.34–7.43)	7.36 (7.30–7.40)	0.922
Base deficit, mmol/L	1.1 (-0.4–2.9)	1.3 (-0.2–3.4)	0.707	1.7 (0.1–3.9)	2.4 (0.6–5.9)	0.676
Lactate, mmol/L	2.40 (1.40–3.20)	2.44 (1.70–2.89)	0.738	2.30 (1.50–3.26)	2.90 (2.00–5.77)	0.454
Laboratory parameters						
White blood cells, × 10^9^/L	8.7 (6.9–12.3)	11.2 (8.9–13.5)	0.013	11.1 (8.9–14.5)	13.7 (10.2–17.9)	0.009
Haemoglobin, g/dL	14.0 (12.8–14.9)	13.1 (11.8–14.3)	0.075	13.9 (12.0–14.8)	12.2 (10.9–13.1)	<0.001
Platelet count, × 10^9^/L	19.1 (15.6–24.1)	17.0 (14.6–21.2)	0.170	23.2 (18.7–26.6)	20.2 (17.0–26.0)	0.079
AST, U/L	29 (24–37)	40 (29-49)	0.022	63 (33–143)	109 (64–196)	0.004
ALT, U/L	24 (17–34)	27 (17–34)	0.813	44 (24–81)	73 (40–116)	0.011
LDH, U/L	249 (208–301)	312 (262–377)	0.001	378 (275–518)	677 (523–1064)	<0.001
CK, U/L	144 (95–232)	175 (115–300)	0.183	289 (183–442)	455 (258–749)	0.001
PT-INR	1.00 (0.96–1.05)	1.10 (0.97–1.17)	0.005	1.02 (0.96–1.07)	1.12 (1.06–1.20)	<0.001
APTT, sec	24.1 (22.5–26.2)	29.8 (25.9-34.1)	<0.001	24.9 (23.1–27.1)	28.1 (24.8–31.9)	<0.001
Fibrinogen, mg/dL	260 (219–299)	207 (149–269)	0.004	248 (206–293)	215 (177–264)	0.014
D-dimer, μg/mL	10.5 (5.8–23.5)	93.2 (52.6–129.3)	<0.001	12.2 (5.8–23.0)	65.0 (54.6–100.0)	<0.001

*ISS* injury severity score, *BP* blood pressure, *AST* aspartate aminotransferase, *ALT* alanine aminotransferase, *LDH* lactate dehydrogenase, *CK* creatine kinase, *PT-INR* prothrombin time–international normalized ratio, *APTT* activated partial thromboplastin time, *TBI* traumatic brain injury

### Haemostatic treatments and amounts of transfusion

The haemostatic treatments and amounts of transfusion in patients with and without hyperfibrinolysis in the two groups are presented in Table 3. Patients with hyperfibrinolysis were transfused larger amounts of red blood cell concentrate and fresh frozen plasma than those without hyperfibrinolysis, in both groups. Moreover, massive transfusions (≥10 units) were frequently performed in hyperfibrinolytic patients in both groups.

**Table 3:** Haemostatic treatments and amounts of transfusion among patients with and without hyperfibrinolysis

	TBI		*P* value	Non-TBI		*P* value
	Hyperfibrinolysis (-) *n* = 77	Hyperfibrinolysis (+) *n* = 34		Hyperfibrinolysis (-) *n* = 80	Hyperfibrinolysis (+) *n* = 46	
Haemostatic intervention, *n* (%)	1 (1)	0 (0)	0.504	28 (35)	25 (54)	0.034
Tranexamic acid administration, *n* (%)	28 (36)	14 (41)	0.630	10 (13)	16 (35)	0.003
Transfusion in the first 6 h after admission						
RBC, units	0 (0–0)	0 (0–4)	<0.001	0 (0–0)	4 (0–12)	<0.001
FFP, units	0 (0–0)	0 (0–6)	<0.001	0 (0–0)	0 (0–10)	0.001
PC, units	0 (0–0)	0 (0–0)	0.168	0 (0–0)	0 (0–0)	0.06
≥ 10 units RCC, *n* (%)	0 (0)	5 (15)	0.001	7 (9)	15 (33)	0.001
Transfusion in the first 24 h after admission						
RBC, units	0 (0–0)	0 (0–8)	<0.001	0 (0–0)	6 (0–16)	<0.001
FFP, units	0 (0–0)	0 (0–6)	<0.001	0 (0–0)	4 (5–12)	<0.001
PC, units	0 (0–0)	0 (0–0)	0.137	0 (0–0)	0 (0–0)	0.05
≥ 10 units RCC, *n* (%)	0 (0)	8 (20)	<0.001	9 (11)	16 (35)	0

*RBC* red blood cell concentrate, *FFP* fresh frozen plasma, *PC* platelet concentrate, *TBI* traumatic brain injury

### Mortality rates

Mortality rates in patients with and without hyperfibrinolysis in the two groups are presented in Table 4. Mortality rates at 24 hours, 48 hours, and 28 days and in-hospital mortality rates were higher in hyperfibrinolytic patients than in those without hyperfibrinolysis in the TBI group only.

**Table 4:** Mortality rates among patients with and without hyperfibrinolysis

	TBI		*P* value	Non-TBI		*P* value
	Hyperfibrinolysis (-) *n* = 77	Hyperfibrinolysis (+) *n* = 34		Hyperfibrinolysis (-) *n* = 80	Hyperfibrinolysis (+) *n* = 46	
Mortality, 24 h, *n* (%)	1 (1)	8 (24)	<0.001	2 (3)	4 (9)	0.12
Mortality, 48 h, *n* (%)	3 (4)	13 (38)	<0.001	2 (3)	4 (9)	0.12
Mortality, 28 d, *n* (%)	4 (5)	17 (50)	<0.001	2 (3)	4 (9)	0.12
In-hospital mortality, *n* (%)	4 (5)	17 (50)	<0.001	2 (3)	4 (9)	0.12

*TBI* traumatic brain injury

### Factors associated with hyperfibrinolysis

**Fig. 2:**
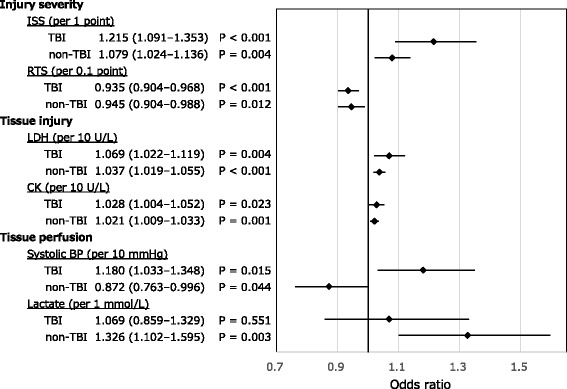
Adjusted odds ratios for hyperfibrinolysis. The odds ratios were adjusted for age and sex. Odds ratios (black squares) and 95% confidence intervals (bars). *TBI* traumatic brain injury, *ISS* injury severity score, *RTS* revised trauma score, *BP* blood pressure, *LDH* lactate dehydrogenase, *CK* creatine kinase

Figure 2 shows the odds ratios for hyperfibrinolysis adjusted for age and sex. Associations between hyperfibrinolysis and injury severity (ISS and revised trauma score) were observed in both the TBI and non-TBI groups. Tissue injury evaluated on the basis of the LDH and CK levels was also associated with hyperfibrinolysis in both groups.

On analysing the relationship between hyperfibrinolysis and tissue perfusion, the systolic blood pressure measurements were associated with hyperfibrinolysis in both groups. However, hyperfibrinolysis was associated with elevated blood pressure in the TBI group, and decreased blood pressure in the non-TBI group. The lactate level, reflecting tissue hypoperfusion, was associated with hyperfibrinolysis in the non-TBI group only.

### Associations between coagulofibrinolytic variables and lactate level

The results of Pearson’s correlation analysis between the coagulofibrinolytic variables and lactate levels in the two groups are presented in Table 5. In the non-TBI group, an increase in the lactate level was correlated with deterioration in each of the coagulofibrinolytic variables (prolongation of prothrombin time (PT) and activated partial thromboplastin time (APTT), decrease in fibrinogen levels, and elevation of D-dimer levels) on arrival at the ED. However, no correlation was observed between the lactate level and any of the coagulofibrinolytic variables in the TBI group.

**Table 5:** Correlations between coagulofibrinolytic variables and lactate levels

	TBI		Non-TBI	
	Pearson’s ρ	*P* value	Pearson's ρ	*P* value
PT-INR	0.182	0.147	0.387	<0.001
APTT	0.054	0.622	0.309	0.001
Fibrinogen	- 0.076	0.525	- 0.202	0.037
D-dimer	0.014	0.902	0.220	0.016

Pearson’s correlation analyses were performed after logarithmic transformation

*PT-INR* prothrombin time–international normalized ratio, *APTT* activated partial thromboplastin time, *DIC* disseminated intravascular coagulation, *TBI* traumatic brain injury

## Discussion

The present study demonstrated that tissue hypoperfusion evaluated on the basis of the lactate level was associated with hyperfibrinolysis and deterioration of coagulofibrinolytic variables (prolongation of prothrombin time (PT) and activated partial thromboplastin time (APTT), decrease in fibrinogen levels, and elevation of D-dimer levels) on arrival at the ED in the non-TBI group, but not in the TBI group. However, hyperfibrinolysis on arrival at the ED was related to injury severity and degree of tissue injury in both the TBI and non-TBI groups.

Previous studies indicate that tissue hypoperfusion may contribute to the development of hyperfibrinolysis, evaluated via thromboelastometry in areas outside the isolated TBI [7–11]. In the present study, hyperfibrinolysis was associated with hypoperfusion and decreased blood pressure in the non-TBI group (Fig. 2). Furthermore, increased lactate levels were correlated with deterioration of the coagulofibrinolytic variables in the non-TBI group. In the fibrinolytic system, one of the key enzymes is t-PA, which catalyses the conversion of plasminogen to plasmin [33]. Tissue hypoperfusion stimulates the endothelial cells and induces the release of t-PA from the endothelial cells into the systemic circulation [34, 35]. Furthermore, this acute and massive t-PA release induces hyperfibrinolysis [2, 7, 8, 10, 36–39]. Previous studies [17, 18] that investigated the relationships between hyperfibrinolysis and hypoperfusion in isolated TBI involved more than twice the number of patients with a base deficit (BD) > 6 mEq/L than that included in the present study. Although the present study was limited to patients with isolated blunt TBI, previous studies also included patients with penetrating brain injury [17, 18]. Moreover, injuries to organs other than the brain were more severe (AIS < 3) than those reported in our study (AIS < 2) [17, 18]. Therefore, the effects of injuries to organs other than the brain and/or penetrating brain injuries should have demonstrated a more pronounced effect on the elevation of BD in the previous studies, when compared with our findings.

We demonstrated that tissue injury, evaluated on the basis of the LDH and CK levels, was associated with hyperfibrinolysis in both the TBI and the non-TBI groups (Fig. 2) in the present study. LDH and CK have been reported to be associated with severity of multiple trauma [40], liver injury [41], TBI [42], and crash syndrome [43]; thus, LDH and CK appropriately represent the degree of trauma-induced cell destruction in various tissues. The destroyed cells release microparticles expressing tissue factor [13, 19, 21–23] and DAMPs [24–26], similar to LDH and CK, into the systemic circulation and activate the coagulation system during the early phase of trauma. The activation of the coagulation system results in fibrin formation and triggers the activation of the fibrinolytic system, thus leading to the generation of plasmin [27]. Consequently, the generated plasmin induces physiological fibrinolysis [27]. However, excessive release of microparticles and DAMPs results in unregulated activation of the coagulation system and formation of massive amounts of fibrin, eventually leading to consumption coagulopathy [13, 19, 21–26]. The massive fibrin formation simultaneously induces increased plasmin generation and the consumption of α_2_-plasmin inhibitor [13, 20, 44]. Low levels of the α_2_-plasmin inhibitor accelerate fibrinolysis and result in hyperfibrinolysis [13, 20, 44].

In the present study, the aetiological factors in hyperfibrinolysis differed between the TBI and non-TBI groups. Both severe tissue injury and hypoperfusion induce hyperfibrinolysis [2, 7, 8, 10, 13, 20, 36–39, 44]. In patients with injuries to the torso (non-TBI group), both severe tissue injury and hypoperfusion are frequent complications. Therefore, hyperfibrinolysis was found to be associated with both severe tissue injury and hypoperfusion in the non-TBI group. On the other hand, in patients with isolated TBI (TBI group), although tissue injury is a common complication, hypoperfusion rarely occurs. Therefore, in the TBI group, hyperfibrinolysis was determined to be associated with tissue injury only, and not tissue hypoperfusion.

Although hyperfibrinolysis in trauma patients is currently evaluated using thromboelastometry [7–11], we used D-dimer as a surrogate marker of hyperfibrinolysis in the present study. Thromboelastometry-indicated hyperfibrinolysis is observed when α_2_-antiplasmin cannot inhibit the action of free plasmin, which is newly activated by the t-PA in the blood sample subjected to thromboelastometry [9]. Raza et al. indicated that hyperfibrinolysis was not detected in more than 90% of trauma patients with marked generation of plasmin using thromboelastometry [9]. Therefore, thromboelastometry can detect hyperfibrinolysis only under the conditions of high free t-PA levels and significantly reduced α_2_-antiplasmin levels [9]. However, elevated D-dimer level is a more sensitive detection tool for hyperfibrinolysis than thromboelastometry, because elevation of the D-dimer level results from degradation of fibrin by plasmin, regardless of the marked increase in free t-PA level and consumption of α_2_-antiplasmin [12]. However, the appropriate cut-off level for D-dimer for diagnosing hyperfibrinolysis is unclear. We adopted ≥ 38 mg/L as the cut-off level for hyperfibrinolysis on the basis of the findings of our previous study, which indicated poor outcomes [3].

Previous studies have investigated the relationship between hyperfibrinolysis and tissue hypoperfusion in severe trauma [2, 7, 9–11, 17, 18]. In these studies, tissue hypoperfusion was assessed on the basis of base deficit [9–11, 17, 18] or lactate level [2, 7, 11]. However, the base deficit value is affected by the balance of sodium, chloride, albumin, and lactate levels [45]. Therefore, in the present study, we used only the lactate level to screen for tissue hypoperfusion because lactate reflects tissue perfusion more directly than does the base deficit [45, 46].

This retrospective multicentre study has several limitations. First, the number of patients with hyperfibrinolysis in the TBI group was not large. Statistical limitations may be involved. Second, the anatomical characteristics of patient injury (e.g., epidural haematoma, subdural haematoma, and cerebral contusion) in the TBI group were unclear. Therefore, the anatomical characteristics of brain injury have not been appropriately reflected in the present analysis. Third, although lactate level was used as a surrogate marker of tissue hypoperfusion in the present study, an increase in the lactate level was not caused only by tissue hypoperfusion, but also by epinephrine-induced β2-adrenoceptor stimulation [46]. In patients with severe trauma, serum epinephrine concentration frequently and significantly increases on arrival at the ED [47]. Therefore, lactate levels in patients with trauma may be affected by the epinephrine released from the adrenal gland due to trauma [46].

## Conclusion

Hyperfibrinolysis is associated with tissue injury in both patients with TBI and in non-TBI patients. However, tissue hypoperfusion is associated with hyperfibrinolysis in non-TBI patients, but not in patients with TBI. Tissue hypoperfusion may therefore not be essential for the development of hyperfibrinolysis in patients with isolated TBI.

## Additional file

Additional file 1: Table S1. Institutional Review Board of each hospital. (ODS 13 kb)

## Abbreviations

AIS: Abbreviated injury score
BD: Base deficit
CK: Creatine kinase
DAMPs: Damage-associated molecular patterns
ED: Emergency department
ISS: Injury severity score
J-OCTET: Japanese Observational Study for Coagulation and Thrombolysis in Early Trauma
LDH: Lactate dehydrogenase
TBI: Traumatic brain injury
t-PA: Tissue-plasminogen activator
